# Ethanol extract of *Chaenomeles speciosa* Nakai induces apoptosis in cancer cells and suppresses tumor growth in mice

**DOI:** 10.3892/ol.2013.1340

**Published:** 2013-05-08

**Authors:** GENDONG YAO, CHAOQI LIU, HONGQI HUO, AIMIN LIU, BAIRUI LV, CAN ZHANG, HAIDONG WANG, JINNONG LI, LIANMING LIAO

**Affiliations:** 1 Department of Biological Therapy, Central Hospital of Handan City, Handan, Hebei 056001;; 2 Molecular and Biology Institute, Three Gorges University, Yichang, Hubei 443002;; 3 Institute of Oncology, Academy of Integrative Medicine, Fujian University of Traditional Chinese Medicine, Fujian, Fuzhou 350102, P.R. China

**Keywords:** *Chaenomeles speciosa* Nakai, immune, apoptosis, cancer, mitochondrial membrane potential

## Abstract

*Chaenomeles speciosa* Nakai is commonly used in traditional Chinese medicine for a variety of health-promoting effects. The present study aimed to investigate the antitumor effects of *Chaenomeles speciosa* Nakai. The tumor-inhibitory activity of the ethanol extract of *Chaenomeles speciosa* Nakai (EEC) was evaluated by *in vitro* growth assays of tumor cells and *in vivo* H_22_ tumor formation assays in mice. Mitochondrial membrane potential and DNA ladder assays were used to detect tumor cell apoptosis in the presence of EEC. To investigate the cellular targets of EEC, the immunomodulatory genes PD-L1, Foxp3 and TGF-β were detected in the tumor tissue using reverse transcription polymerase chain reaction (RT-PCR). Immune responses were determined by hemolysis and lymphocyte proliferation assays. EEC markedly inhibited the proliferation of the H_22_ cells in a dose-dependent manner. Moreover, it induced DNA fragmentation and decreased the mitochondrial membrane potential. *In vivo*, EEC inhibited tumor growth and enhanced the immune responses in mice, while the expression of PD-L1, Foxp3 and TGF-β was inhibited in the tumor tissue. These results provide the first evidence that EEC may inhibit tumor growth by directly killing tumor cells and enhancing immune function. Thus, it is a natural source for safe anticancer medicine.

## Introduction

*Chaenomeles speciosa* Nakai (*C. speciosa* Nakai) has been used in traditional Chinese medicine for thousands of years to treat a variety of diseases, including sunstroke, edema and arthralgia. During the past decades, *C. speciosa* Nakai has been employed to treat diarrhea (1) and hepatitis (2). More recently, *C. speciosa* Nakai has also been used to treat arthritis (3–5). Studies have revealed that *C. speciosa* Nakai has antioxidant and immunomodulatory properties (6,7).

In addition, Chinese herbalists have used *C. speciosa* Nakai to treat cancer, particularly hepatocellular carcinoma. Curative treatments, including surgery, local destruction techniques and liver transplantation, for hepatocellular carcinoma (HCC) are only achievable in certain patients. The majority of patients must undergo chemotherapy. Although chemotherapy has been shown to improve the survival of patients with HCC, there are several serious side-effects and drug resistance may occur. These two obstacles to more successful therapeutic outcomes have been major challenges for oncologists. The challenge of identifying new therapeutic approaches that alleviate the adverse effects of chemotherapy, while also improving their efficiency, is clear and urgent. Consequently, the present study investigated whether *C. speciosa* Nakai extracts may be used for the treatment of HCC. The present study investigated the effects of the ethanol extract of *C. speciosa* Nakai (EEC) in inhibiting tumor growth *in vivo* and *in vitro*.

## Materials and methods

### Materials

RPMI-1640 medium and fetal bovine serum were obtained from Invitrogen (Carlsbad, CA, USA). Unless stated otherwise, all other chemicals were obtained from Sigma-Aldrich (Zwijndrecht, the Netherlands).

### Preparation of EEC

A total of 2 kg field-raised, air-dried *C. speciosa* Nakai was ground to a fine powder and added to 4,000 ml ethanol [final ethanol concentration 60% (w/v)] for extraction for 1 h at 75°C. The extract was incubated in a rotary evaporator until all of the ethanol had evaporated. The extract was then solubilized in saline and used for the present experiments. Overall, 1 ml solution contained extract that was equal to 0.2 g raw *C. speciosa* Nakai.

### In vivo tumor model

Kunming mice were obtained from the Hubei Laboratory Animal Center (Wuhan, China). The mice had free access to pellet food and water. The animals were acclimated to laboratory conditions at a temperature of 18–25°C, with a relative humidity of 55 to 65% and a 12-h light/dark cycle, for one week prior to the experiments. All animal experiments were approved by the Institutional Animal Control and Utilization Committee of the Central Hospital of Handan City.

The murine H_22_ cell line was obtained from the School of Basic Medical Science, Peking University (Beijing, China). Cells were cultured in Dulbecco’s modified Eagle’s medium (Gibco Laboratories, Grand Island, NY, USA) supplemented with 10% fetal bovine serum, penicillin and streptomycin (10 U/l, Gibco Laboratories) in a humidified atmosphere with 5% CO_2_ at 37°C. The hepatoma model was established by the subcutaneous inoculation of H_22_ cells (1×10^6^ cells per mouse) into the right flank of the mice (8). At 48 h post-inoculation, the mice were randomly divided into three groups: i) the vehicle-treated control group, which received 0.5 ml saline by gavage; ii) the EEC group, which received 0.5 ml EEC; and iii) the cisplatin group, which received 5 g/kg cisplatin intraperitoneally. Another eight mice, which were not inoculated with H_22_ cells, served as normal controls.

Red blood cells (RBCs; 5% 0.2 ml per mouse) were injected into the peritoneal cavity on day 14. All the mice were sacrificed at day 18 and the tumors were excised and weighed. The tumor inhibition rate was calculated according to the formula: inhibition rate (%) = (1 − tumor weight in test group / tumor weight in control) × 100.

### Hemolysis assay

Sheep RBCs (20%) were prepared with natural saline. The RBCs (0.2 ml 20%) were injected intraperitoneally into the mice. After four days, the specific IgM of the serum was detected as previously described (9). Briefly, 100 *μ*l 50X diluted mouse serum, 50 *μ*l 10X diluted complement and 50 *μ*l 5% sheep RBCs were added to the ELISA plate and incubated for 30 min at 37°C. Following centrifugation, 150 *μ*l supernatant was transferred to a 96-well plate to detect the absorbance at OD_415_. The 50% hemolysin value (HC_50_) was calculated as follows: HC_50_ = sample dilution × OD_sample_ / (OD_RBC_ / 2). OD_RBC_ was the OD value of the complete lysis of the RBCs.

### Flow cytometry

Splenocytes were prepared and cultured in 24-well plates for 48 h, then the cells were washed and fixed in 70% alcohol overnight at 4°C. The cells were washed with PBS and 500 *μ*l RNase (30 *μ*l/ug) was added into Eppendorf tubes for 30 min. Finally, propidium iodide dye was added in a dark room (10,11) and the cell cycle was analyzed using flow cytometry (FCM). The lymphocyte proliferation rate (%) was calculated as follows: proliferation rate (%) = [(S + G_2_M) / (G_0/1_ + S + G_2_M)] × 100.

### Reverse transcription-polymerase chain reaction (RT-PCR)

The H_22_ tumor tissues were collected and the total RNA was isolated using the RNase mini kit (Invitrogen). First-strand cDNA was synthesized from the total RNA with Super Script II reverse transcriptase (Invitrogen) using oligodT as the reverse primer.

The cDNA was amplified by PCR in a programmable DNA thermal cycler for 30 cycles (95°C for 3 min, 95°C for 30 sec, 55°C for 30 sec and 72°C for 30 sec). The primer sequences used for the PCR are shown in Table I.

### Proliferation assay

The percentage of growth inhibition was determined with the 3-(4,5-dimethylthiazol-2-yl)-2,5-diphenyltetrazolium bromide (MTT) assay (12). Hepatocellular carcinoma H_22_ cells (4,000 cells/well) were seeded into 96-well plates and treated with EEC. The cells were incubated overnight at 37°C with 5% CO_2_. Subsequently, 100 *μ*l MTT, at a concentration of 2.5 mg/ml, was added to each well and the cells were incubated for an additional 4 h. The supernatant was then aspirated and dimethyl sulfoxide was added to the wells to dissolve the precipitate. The absorbance was determined at 570 nm using a micro-plate reader (Multiskan Spectrum; Thermo Electron Corporation, Vantaa, Finland). The cell survival rate (%) = 100 × O_DEEC group_ / OD_control group_.

### DNA fragmentation assay

The H_22_ cells were cultured in the presence or absence of EEC for 48 h, then gently scraped and harvested by centrifugation. The cells were incubated for 20 min in DNA fragmentation lysis buffer [50 mmol/l Tris-HCl (pH 8.0), 20 mmol/l ethylenediaminetetraacetic acid and 0.5% Triton X-100] on ice. The cells were then centrifuged at 12,000 × g for 30 min.

DNA was extracted with a mixture of phenol and chloroform (1:1) and precipitated with twice the volume of cold ethanol and sodium acetate. The precipitates were resuspended in 10 *μ*l 10 mM Tris (pH 7.8) and 1 mM EDTA buffer and incubated for 30 min at 37°C with 1 *μ*g/ml RNase (Roche Molecular Biochemicals, Indianapolis, IN, USA) to remove the RNA. The DNA pellets were electrophoresed for 90 min at 100 V on 2% agarose gels. The gel was stained with ethidium bromide and the DNA fragments were visualized under ultraviolet light.

### Measurement of mitochondrial membrane potential (Δψm)

The H_22_ cells (2×10^4^ cells/well) were seeded into a 96-well plate and incubated overnight at 37°C, with 5% CO_2_. Subsequently, the cells were treated with various concentrations of EEC and incubated for an additional 10 h (13). To measure Δψm, the cells were incubated with DiOC6 (100 nM; Molecular Probes, Eugene, OR, USA) during the last 30 min of treatment. DiOC6 is a fluorescent dye that is incorporated into the mitochondria in a Δψm-dependent manner. Once the cells had been sufficiently washed, the fluorescence intensity was determined using a multifunctional micro-plate reader (14).

### Statistical analysis

All values are expressed as the mean results of the triplicate experiments ± standard deviation. The unpaired Student’s t-test was used to identify differences between the groups. P<0.05 was considered to indicate a statistically significant difference. SPSS 10.0 (SPSS, Inc., Chicago, IL, USA) was used for the statistical analyses.

## Results

### EEC has marked antiproliferative and apoptotic effects in cancer cells

**Figure 1.**
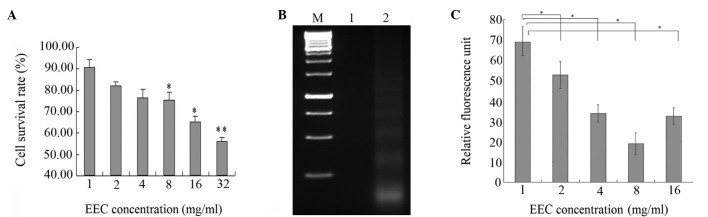
Antiproliferative and apoptotic effect of EEC on cancer cells. (A) H_22_ cells (5×10^4^) were plated in 96-well culture plates. Subsequent to 24 h, the medium was changed to fresh medium and treated with saline alone or EEC at the indicated doses. Following 48 h of treatment, the cells were analyzed by MTT assay. (B) The effect of EEC on the DNA fragmentation of the H_22_ cells. Ladders were detected by 1.5% agarose gel electrophoresis. M, 1K DNA marker; lane 1, untreated H_22_ cells; lane 2, H_22_ cells treated with EEC. (C) Following 30 min of incubation with DiOC6 (100 nM) for Δψm, the intracellular fluorescence intensity was measured. Results represent the mean ± SEM from three independent experiments. ^*^P<0.05 vs. control; ^**^P<0.01 vs. control. EEC, ethanol extract of *Chaenomeles speciosa* Nakai; Δψm, mitochondrial membrane potential.

The effect of EEC on the growth of cancer cells is expressed as the percentage of cell viability relative to the control. As shown in Fig. 1A, EEC inhibited the proliferation of the H_22_ cells in a dose-dependent manner. Compared with the saline-treated controls, EEC at a dose of 32 mg/ml caused 57.5% inhibition of H_22_ cell proliferation (P<0.001). The cells treated with EEC showed typical apoptotic morphologies, including cell shrinkage and rounding and cell membrane blebbing, as well as nuclear fragmentation and condensation (data not shown). Apoptosis was characterized by the activation of endogenous endonucleases with the subsequent cleavage of chromatin DNA into internucleosomal fragments of 180 to 200 bp. The extracted cell DNA was detected by agarose gel electrophoresis. Fragmented DNA was clearly observable in the H_22_ cells following treatment with EEC for 48 h, whereas the cells did not produce ladders without treatment. Thus, the apoptotic effect of EEC on the tumor cells was further demonstrated by DNA fragmentation (Fig. 1B).

Mitochondrial damage is significant in cell apoptosis. Therefore, to determine whether Δψm is involved in the regulation of apoptosis induced by EEC, the fluorescent lipophilic cation DiOC6 was used as an indicator of the energy state of the mitochondria. As shown in Fig. 1C, EEC treatment led to a rapid drop in mitochondrial energy, as demonstrated by a decrease in fluorescence from the baseline following 12 h of treatment (M1, 47.55% in the control cells vs. 77.43% in the EEC-treated H_22_ cells; the Δψm peak shifted to the left, indicating that fewer cells retained DiOC6 in their mitochondria). These results suggested that the inhibitory effect of EEC on tumor cell growth may be through the induction of apoptosis.

### EEC inhibits tumor growth in vivo

The antitumor activity of EEC was investigated in the H_22_ murine hepatoma model. The results showed that EEC and cisplatin significantly inhibited tumor growth compared with the vehicle-treated group, with inhibitory rates of 39.82±4.98 and 58.33±9.29%, respectively (P<0.05 compared with vehicle-treated group; Table II). These data indicate that EEC has the ability to inhibit the growth of H_22_ cells *in vivo*.

The gross toxicity of EEC and cisplatin was then compared using the body weights of the mice. The body weights were 19–21 g at baseline and were similar among the three groups. Subsequent to 18 days of treatment the differences in net body weight per mouse (body weight - tumor weight) were statistically significant among the three groups (Table II). The net body weight of the EEC group was significantly higher than that of the cisplatin group, indicating that EEC has less severe adverse reactions compared with cisplatin.

### EEC enhances immunity in mice

To further investigate the mechanism by which EEC inhibits tumor growth, lymphocyte proliferative activity was evaluated using a flow cytometry assay. As shown in Table III, the cell cycle analysis showed that EEC enhanced lymphocyte proliferation. Moreover, the hemolysis assay showed that EEC significantly increased the production of RBC antibody (HC_50_; Table III). Compared with the vehicle-treated group, cisplatin significantly decreased the production of RBC antibody. These data indicate that EEC inhibits tumor growth partially via enhancing host immunity.

### EEC suppresses the expression of PD-L1, Foxp3 and TGF-β

**Figure 2.**
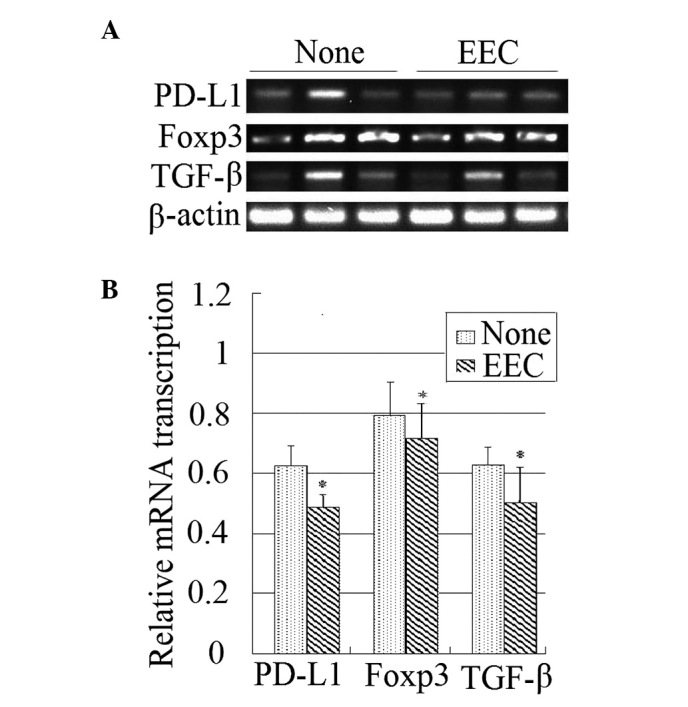
(A) Agarose gel electrophoresis of reverse transcription polymerase chain reaction products. (B) Gel bands in the digitized image were selected and their average gray-scale density measured. The results were compared between groups. ^*^P<0.05 vs. control. EEC, ethanol extract of *Chaenomeles speciosa* Nakai.

PD-L1 is a ligand of programmed death 1 (PD-1), which is expressed on activated lymphocytes and negatively regulates the immune response. Foxp3 is specifically expressed on regulatory T (Treg) cells and suppresses effective lymphocyte activity. TGF-β is a cytokine that activates the Treg cells. All of these factors are negative immunomodulatory factors. The present study observed that EEC decreases the expression levels of these three genes in the tumor tissues, as indicated by RT-PCR (Fig. 2).

## Discussion

The present study demonstrated that EEC inhibits the growth of H_22_ cells *in vivo* and causes the apoptosis of cells *in vitro*. Furthermore, tumors isolated from the inoculated mice expressed more PD-L1, Foxp3 and TGF-β in the vehicle-treated group compared with the EEC group, suggesting that EEC is able to inhibit the expression of negative immnoregulatory genes.

Despite advances in the understanding of the carcinogenic processes of cancer, the relatively low remission rate of chemotherapy, radiotherapy and immunotherapy have encouraged the scientific community to identify active medicinal compounds from herbal/natural sources. In the present study, it was demonstrated that EEC was able to suppress cancer cell growth through the induction of apoptosis, the inhibition of cell proliferation and the promotion of host immune function. This is the first study that clearly characterizes the antitumor properties of EEC in cancer cells and tumor xenografts.

It is not clear which compounds are responsible for the anti-tumor effects of EEC. Triterpenoids, flavonoids and organic acids are the main classes of compounds identified from *C. speciosa* Nakai (15–20). The major triterpenoids include 3-O-acetylursolic acid, 3-O-acetylpomolic acid, oleanolic acid and betulinic acid. Betulinic acid was initially characterized as a highly selective inhibitor of human melanoma cells and tumor growth through the induction of apoptosis (21). Subsequent research has shown that betulinic acid is an effective inhibitor of cell proliferation and that it induces apoptosis in numerous types of cancer cells (22). Oleanolic acid has also been shown to induce apoptosis in leukemia cells (23–26). Therefore, the antitumor effects of EEC may involve contributions by oleanolic acid and betulinic acid.

In order to further clarify the mechanism involved in the EEC-induced growth suppression of tumor xenografts, the immune function of tumor-bearing mice was investigate in the present study. EEC was observed to promote the production of immunoglobulin. Further investigation demonstrated that EEC was able to suppress the expression of PD-L1, Foxp3 and TGF-β. The transcription factor Foxp3 has a major role in the development of Treg cells and is critical for their suppressive function (27). Naive CD4^+^ T cells selected by Foxp3 during development in the thymus have the potential to be converted into functional Foxp3^+^ Treg [adaptive or induced Treg (iTreg)] cells in peripheral lymphoid organs or tissue culture. Thus, the decreased expression of Foxp3 in the EEC-treated tumors may indicate that there are fewer Treg cells in EEC-treated tumors compared with vehicle-treated tumors. In accordance with this finding, the expression of TGF-β, a factor that is critical in the induction of Foxp3 expression *in vitro* and *in vivo*, was also suppressed. Furthermore, the expression of PD-L1, the ligand of PD-1, was also suppressed by EEC. PD-1 is an inhibitory receptor that is expressed on activated lymphocytes and that regulates tolerance and autoimmunity. These findings suggest that the enhancement of immunity in EEC-treated tumor-bearing mice may be associated with the suppression of immune tolerance.

In addition, the promising antitumor effect of EEC was achieved without the toxicity and side-effects anticipated with cisplatin (e.g. a significant drop in body weight and immunosuppression), which further suggests that EEC has the potential to be established as novel adjuvant agent in cancer chemotherapy. At present, the fractionation of EEC is being performed to identify the fractions that contain the bioactive constituents responsible for the growth-inhibitory effects.

In summary, the present study demonstrated that EEC was able to inhibit cancer cell growth *in vitro* and *in vivo*. In contrast to orthodox chemotherapy using cytotoxic drugs, the use of this herbal extract imposes less toxicity while retaining antitumor effects. This indicated the possibility of further developing EEC as an adjuvant chemotherapeutic agent in cancer therapy.

## Figures and Tables

**Table I.** Oligonucleotide primers.

Gene	Primer sequences (5′-3′)
PD-L1	AGG CAA GCT TAT GTG GGT CCG GCA GGT AC
	AGG CGA ATT CTC AAA GAG GCC AAG AAC AAT
Foxp3	CCC TTT CAC CTA TGC CAC CCT
	GCT CCC TTC TCG CTC TCCAC
TGF-β	ACG GCA TGG ATC TCA AAG AC
	GTG GGT GAG GAG CAC GTA GT
β-actin	TCA CCC ACA CTG TGC CCC ATC TAC GA
	CAG CGG AAC CGC TCA TTG CCA ATG G

**Table II.** Inhibitory effect of EEC on H_22_ murine hepatoma cell growth in mice.

Group	Body weight (g)	Tumor weight (g)	Net body weight (g) (body weight − tumor weight)	Tumor inhibition rate (%)
Vehicle-treated	32.91±5.11	1.73±0.32	31.18±4.79	-
EEC	29.00±4.63	1.04±0.13	27.96±4.52^a^	39.82±4.98
Cisplatin	19.28±2.41	0.72±0.12	18.56±2.29^a^	58.33±9.29

Data are presented as mean ± standard deviation.

a P<0.05 vs. vehicle-treated group. EEC, ethanol extract of *Chaenomeles speciosa* Nakai.

**Table III.** Antibody production and inhibition of lymphocyte proliferation.

Group	Proliferation rate (%)	RBC antibody (HC_50_)
Normal	63.75±8.93	179.80±22.47
Vehicle-treated	49.18±19.23	110.34±13.79
EEC	55.20±16.82^a^	188.44±23.56^b^
Cisplatin	39.78±19.79	54.32±6.79

Data are presented as mean ± standard deviation.

a P<0.05;

b P<0.01 vs. cisplatin. EEC, ethanol extract of *Chaenomeles speciosa* Nakai.
